# Rates of Adsorption and Desorption of Polystyrene on Chrome Surface

**DOI:** 10.6028/jres.068A.038

**Published:** 1964-08-01

**Authors:** Robert R. Stromberg, Warren H. Grant, Elio Passaglia

## Abstract

The rate of adsorption of polystyrene from cyclohexane solution on chrome ferrotype plates was studied for a concentration range of 10^−1^ to 10^−4^ mg/ml. Two molecular weight fractions of polymers, 76,000 and 38,100, were prepared by the anionic polymerization of styrene tagged with tritium, and a radiotracer technique was used to measure directly the amount of polymer adsorbed on the surface. The rate of adsorption is very dependent on the concentration of the polymer solution, and times varying from minutes to several hours were required before maximum adsorption occurred for the concentration range studied. The rate of desorption is strongly dependent on the adsorbance; it was hypothesized that this is due to the number of attachments per molecule also varying with adsorbance. The conformation of the adsorbed molecule as indicated by these results and those determined by the measurement of the thickness of the adsorbed layer by ellipsometry is discussed.

## 1. Introduction

Pointer adsorption is most frequently studied by measuring changes in solution concentration after adsorption on a relatively high surface area solid, such as a powder. To achieve sensitivity in such measurements, the change in solution concentration must be relatively large, and the adsorption would therefore occur over a considerable solution concentration range. It has been well established that the rate of desorption of polymers can in some cases proceed so slowly that the appearance of an irreversible process is created [e.g., 1, 2, 3].^1^ The results of adsorption experiments that begin at a high concentration and end at a lower concentration may not represent equilibrium values.

It is desirable, therefore, to carry out measurements in which the solution does not change significantly in concentration. This is possible with the radiotracer technique. Measurements can be made directly on the adsorbent surface with no significant change occurring in solution concentration. Because of the sensitivity of the method, it is also possible to make measurements from much more dilute solutions than is usually possible by most other means.

This paper will report the results of a study of the rates of adsorption and desorption of polystyrene with narrow molecular weight distributions from cyclohexane solution on chrome ferrotype surface. The use of labeled polystyrene permitted the accurate determination of the rate of adsorption over a relatively large range of solution concentrations, the adsorption isotherm at low concentrations, and the desorption rate in a direct manner.

## 2. Specific Activity of Polymer and Counter Efficiency

The specific activity of the polymer was determined in a liquid scintillation counter using NBS standard tritiated water sample No. 4927 as a standard. The activity of this sample was 1.33×10^6^ dps/ml as of August 20, 1954. The liquid scintillation counter was calibrated by adding a 10:1 dilution of the standard tritium oxide in quantities ranging from 6 mg to 14 mg, to 20 ml portions of a scintillating solution. This solution was made up of toluene and ethanol in a 7: 3 ratio and contained 3.5 percent of 2,5-diphenyloxazole-(PPO) and 0.35 percent of 1,4-bis-2(4-methyl-5-phenyloxazolyl) benzene-(Dimethyl POPOP). Quantities of polystyrene in toluene ranging from 38 to 120 mg were also added to the scintillating solutions. The specific activity of the polystyrene was determined to be 4.65 millicuries per gram.

The determination of the quantity of radioactive polystyrene adsorbed on metal was carried out by counting metal slides covered with adsorbed polymer in a 2*π* windowless gas-flow Geiger counter. The efficiency of the counter was determined in the following manner. It was assumed that the adsorbed polymer would result in an “infinitely thin film” which would have very little self adsorbance of the *β* radiation. It was necessary, therefore, to prepare very thin films of known amounts of polymer. This was done by preparing polymer solutions, 10^−2^ end 10^−3^ mg/ml in benzene, and transferring quantities ranging from 10^−2^ to 10^−3^ ml of these solutions into chrome saucers containing about 1 ml of chloroform to enhance the spreading of the polymer on the metal surface. The shape of the saucer was such that only a very shallow cavity was formed, and the entire surface was available to the counter with no shielding by edges. Quantities ranging from 10^−4^ to 2×10^−6^ mg were placed in these vessels and counted.

It is realized that this polymer would not dry into a continuous film, but rather that some drying pattern, invisible to the eye, would be formed and that there would be some self adsorption at thick areas. The measurements were carried out, therefore, for the series of quantities given above and the results extrapolated to zero concentration. The slope of the line was small, and the efficiencies ranged from 53 percent for the largest amount on the saucer to 60 percent for the extrapolated value at zero dilution. Inasmuch as it is believed that experimental error in the determination of the counter efficiency would result in measured efficiencies that are lower than the actual efficiency for the adsorption experiments, the extrapolated value of 60 percent was used. As the geometry of the counter was 2*π*, a backscatter of at least 20 percent contributed to the total measured count.

## 3. Experimental

### 3.1. Polymer Preparation and Characterization

The polystyrene was prepared by the anionic polymerization of styrene that had been labeled with a tritium atom attached to the C-8 position. The polymerization was carried out in a manner described by Wenger and Yen [4]. The initiator was prepared by mixing under vacuum known quantities of butyl lithium, nonlabeled styrene, and benzene, and was transferred to calibrated tubes. The polymerization was also carried out under vacuum using labeled styrene previously dried over calcium hydride and vacuum distilled. The benzene solvent was also dried over calcium hydride and then vacuum distilled from initiator solution which had been previously used to rinse the polymerization vessel. The labeled styrene was titrated with a dilute initiator solution to deactivate impurities prior to addition of the initiator used for the polymerization.

The polymer was fractionated by conventional precipitation techniques and eight molecular weight fractions were obtained. These fractions were grouped very close to either a viscosity molecular weight of 76,000 or a molecular weight of 38,000. Two fractions with molecular weights of 76,000 and 38,100 were used in this study. The two fractions studied were examined by the ultracentrifuge.^2^ Each fraction contained a small amount of the other fraction.

### 3.2. Adsorbent Surface

Chrome ferrotype plate was used as the adsorbent surface. Immediately prior to use 2×2 cm slides were cleaned by immersion in warm chromic acid-sulfuric acid cleaning solution and washed thoroughly in hot distilled water. They were then passed several times through a warm flame, and while still warm immersed in the solvent, cyclohexane. The surface areas used here are based on a geometric projection. The actual surface areas were somewhat higher because of surface roughness and, therefore, the reported adsorbances (amount adsorbed per unit area) are somewhat high.

### 3.3. Adsorption Procedure

The concentration range 10^−1^ to 10^−4^ mg/ml of polystyrene in cyclohexane was studied. The adsorption vessels consisted of glass jars with tightly fitting covers that were lined with polytetrafluoroethylene. Approximately 50 ml of solution was contained in the jars. It was necessary to precondition containers such as volumetric flasks, adsorption vessels, etc., for the more dilute polymer solutions, as sufficient polymer would be adsorbed on the container walls to bring about a significant decrease in the solution concentration. The vessels were preconditioned by repeated exposure to fresh polymer solution until there was no change in solution concentration with time as determined by liquid scintillation counting. Prior to adsorption, the solutions and slides were kept in a bath maintained at 30 °C. The slides were removed from the solvent, drained and placed in the solution for a predetermined period of time. During this time, the adsorption vessel together with its slides was shaken vigorously in a bath at 30 °C.

After this interval, the slide was removed from the solution, quickly drained, dipped in and out of solvent, dried, and counted. The solvent rinse was required to remove polymer carried out with the excess solution. The time interval in the solvent was less than 1–2 seconds. Increasing this time did not change the amount of polymer on a slide, and the reproducibility by this technique was very good. When the solvent rinse was eliminated, the reproducibility from sample to sample was very poor, especially with the more concentrated solutions.

Two different techniques were used to obtain the adsorption results. In one, a new set of slides was used for each time interval. All such measurements were run in triplicate. In the second technique, the same slide was exposed to the polymer solution for different periods of time, i.e., counted and then reimmersed in the polymer solution for additional periods. It was possible by means of this second technique to follow the adsorption on a specific slide. These measurements were carried out in quadruplicate, with each slide individually followed.

### 3.4. Desorption Procedure

Slides were immersed in the polymer solution for a predetermined period of time at 30 °C, drained, very quickly rinsed in solvent and placed in another jar containing solvent only. The slides were allowed to remain in the solvent at 30 °C for a predetermined period, drained, dried, and counted. Again, as in the case of adsorption, two separate techniques were followed. In one, different slides were used for each desorption time, again the measurements being made in triplicate. For the other, adsorption was carried out as described above, the slides were drained, counted, placed in the solvent for a predetermined time, drained, counted, and again replaced in the solvent for additional periods. These desorption runs were made in quadruplicate, again with each slide being followed individually.

### 3.5. Counting Procedure

The 2×2 cm slides were placed in a planchette, covered with a mask with an available counting area of 2.27 cm^2^ and counted in a windowless Geiger counter equipped with an automatic changer. The counting area was, therefore, constant and the edges of the slide were not counted. The samples were counted for a total count of 5120, which was accomplished in two steps of 2560 each, with the mask being removed and replaced after one-half of the counting had been completed. The background was determined daily and averaged about 48 counts/minute.

## 4. Results

### 4.1. Adsorption

**Figure 1:**
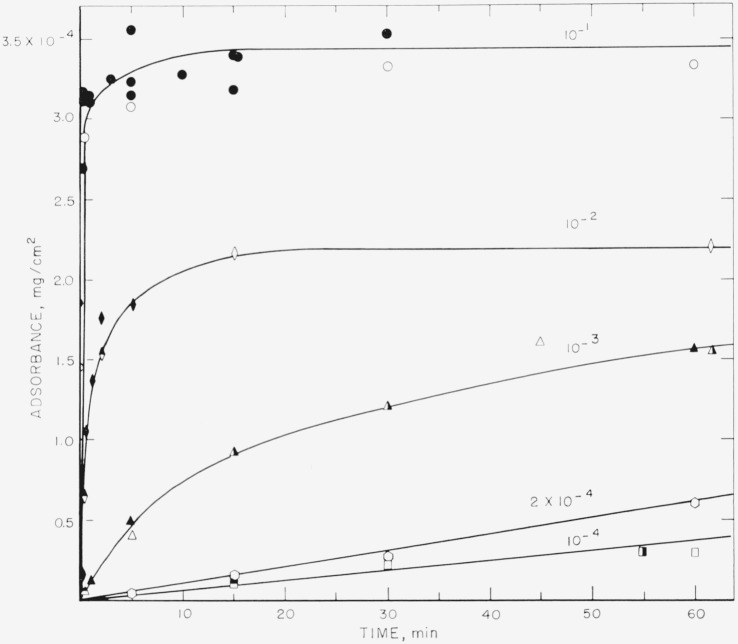
Adsorbance of polystyrene, mol wt = 76,000, versus time for intervals up to 1 hr. Solution concentration

**Figure 2:**
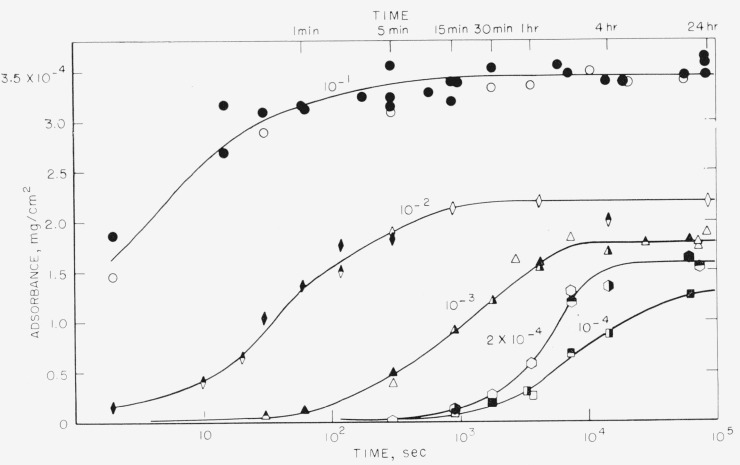
Adsorbance of polystyrene, mol wt = 76,000 versus log time. Solution concentration

**Figure 3:**
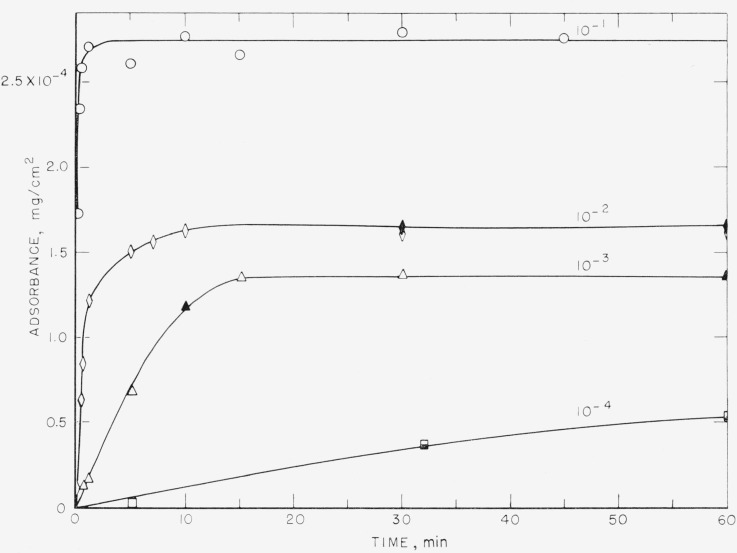
Adsorbance of polystyrene, mol wt = 38,100 versus time for intervals up to 1 hr. Solution concentration

**Figure 4:**
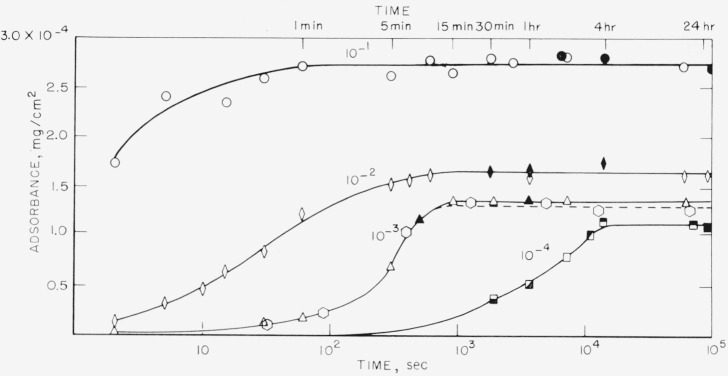
Adsorbance of polystyrene, mol wt = 38,100 versus log time. Solution concentration

The amount adsorbed per unit area, called adsorbance, in mg/cm^2^, is plotted as a function of time in figures 1 to 4 for the polystyrene samples studied. These results were obtained from solutions of polymer in cyclohexane ranging in concentration from 10^−1^ to 10^−4^ mg/ml. The results for the 76,000 molecular weight sample are given in figures 1 and 2, and for the 38,100 molecular weight sample in figures 3 and 4. Figures 1 and 3 are plotted as adsorbance versus time for the first hour of adsorption time. The curves in figures 2 and 4 are plotted as adsorbance versus log time for the entire period studied. Except for the dashed curve in figure 4, all points represent the average of three slides and the various shadings of a specific symbol represent different runs. All of the points on figures 1–4, except for the dashed curve on figure 4, were obtained using different slides for each point. The curves are labeled to correspond to the solution concentration.

Comparison of these four figures shows that the time required to attain a plateau is very dependent on both solution concentration and molecular weight. For the 76,000 molecular weight sample, the times required varied from approximately 5 min for the 10^−1^ mg/ml concentration to about 24 hr for the 10^−4^ mg/ml solution. For the lower molecular weight sample, this time was reduced to about 1 min for the 10^−1^ mg/ml concentration and about 4 hr for the 10^−4^ mg/ml solution. The times required for equilibrium were also shorter for the intermediate concentrations, approximately 15 min being required for the 38,100 molecular weight sample from the 10^−3^ mg/ml solution, compared to about 2 hr for the higher molecular weight sample.

The dashed line in figure 4 represents the curve obtained when the same slides were repeatedly reimmersed in polymer solution of a concentration 10^−3^ mg/ml, after having been removed and counted. The points represent the average of 4 slides. The times give the cumulative total time the slides were exposed to the solution. It is observed that there is very little difference between the curve obtained using different slides for each exposure time and that obtained by re-exposing the same slides.

**Figure 5:**
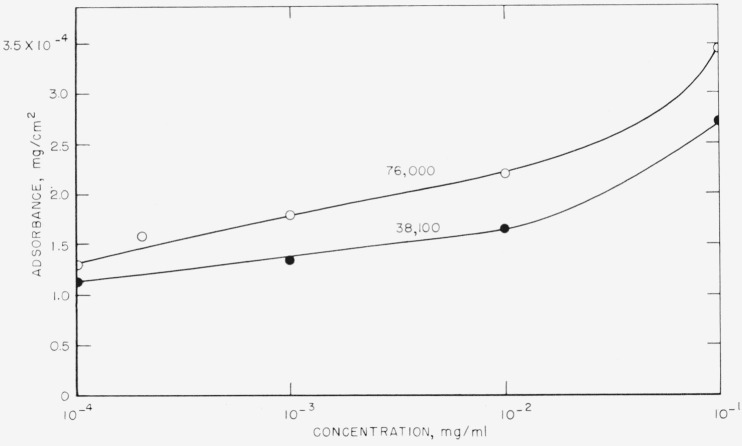
Adsorption isotherm of polystyrene for range of concentrations studied. ○ Molecular weight = 76,000 ● Molecular weight=38,100

The adsorbance attained at equilibrium for each of the concentrations studied is plotted versus the log of concentration in figure 5 for both molecular weight samples. It is readily observed that a plateau in the adsorption isotherm is not reached for either of these molecular weights, and that a concentration in excess of 10^−1^ mg/ml is required to attain the maximum absorbance.

### 4.2. Desorption

**Figure 6:**
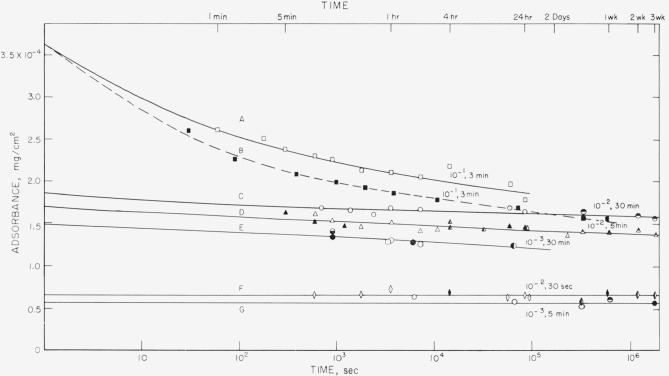
Desorption of polystyrene, mol wt = 76,000 versus log time. Curve Solution concentration and time of immersion prior to desorption in solvent, cyclohexane

**Figure 7:**
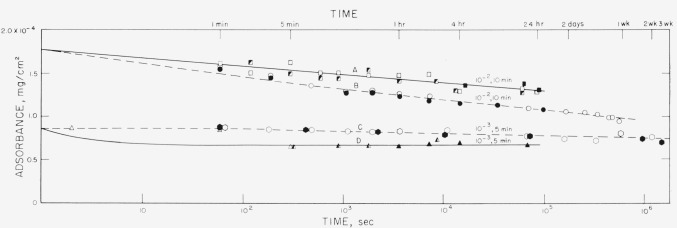
Desorption of polystyrene, mol wt = 38,100 versus log time. Curve Solution concentration and time of immersion prior to desorption in solvent, cyclohexane

The results of the desorption of polystyrene, molecular weight 76,000, into solvent is shown in figure 6 in which the amount of polymer remaining on the slide is plotted against the log of the time of exposure of the slide to the solvent, cyclohexane. Similar curves are given in figure 7 for the lower molecular weight material.

Curves A, C, D, E, F, and G in figure 6 and curves A and D in figure 7 represent exposure of slides for a predetermined time to solutions with concentrations which are given in the captions, followed by rinsing and placement in pure solvent. The slides were not allowed to dry in this process. Different slides were used for each time interval. The various shadings for each symbol represent different runs. The initial values are the averages of several measurements made during the course of a run and of separate runs.

Curve B in figure 6 and curves B and C in figure 7, represented by dashed lines, were obtained by reexposing the same slides to solvent for different time periods. After each exposure they were dried, counted, and reimmersed in solvent. Each point represents the average of 4 slides. The closed and open points on curves B and C in figure 7 represent different runs, i.e., two different sets of slides. The differences between curves A and B in figure 6, A and B in figure 7 and C and D in figure 7 are presumably caused by drying of the adsorbed film prior to desorption. The drying process and subsequent rearrangement of the polymer molecule may cause the disruption of some adsorbed bonds, resulting in fewer attachments per molecule and consequently more rapid desorption. Of course, time may also be required to reswell the “collapsed” film. Although curve C in figure 7 is slightly higher than curve D, except for the initial desorption shown by curve D, both curves are very similar, and these measurements may not be sufficiently sensitive to detect small changes at low adsorbance values.

The rate of desorption into solvent depends on both the molecular weight of the adsorbed polymer and its initial adsorbance. As shown by curves A in figure 6 for the largest initial adsorbance studied, desorption proceeded initially quite rapidly, but then at a continuously decreasing rate, for the 76,000 molecular weight sample. For the case of lower initial adsorbance as seen in curves C, D, and E, figure 6, desorption proceeded at a slower rate, but in a constant manner for the entire period studied, which in some cases was as long as 3 weeks. At still lower initial adsorbance, no measurable desorption occurred, again for periods as long as 3 weeks, as seen in curves F and G, figure 6.

The lower molecular weight polymer desorbed at a more rapid rate than the higher molecular weight sample, at comparable initial adsorbance. This is illustrated by comparing curves C and D in figure 6 with curve A in figure 7. The initial adsorbance values for curves C and D, figure 6, are respectively slightly higher and slightly lower than that for curve A, figure 7. However, the rate of desorption of the 38,100 molecular weight sample, curve A, is greater than that of the 76,000 molecular weight polymer, curves C and D. As in the case of the higher molecular weight sample, the rate of desorption is decreased when the initial adsorbance is decreased. This is shown by curve D, figure 7, in which after an initial desorption, no additional desorption could be detected for the time intervals studied.

The rate of desorption does not appear to depend on the concentration of the original adsorption solution, nor on the time of exposure to that solution, but only on the final adsorbance for a given molecular weight. The initial adsorbance values for curves C, D, and E in figure 6 are all fairly close together. Curves C and D were obtained after adsorption for a solution with a concentration of 10^−2^ mg/ml for 30 min and 5 min, respectively. Curve E was obtained after adsorption for a 10^−3^ mg/ml solution for 30 min. The slopes of all three curves are quite similar, although the adsorption conditions were different.

## 5. Discussion

The adsorption of macromolecules, in many respects, is different from the adsorption of small molecules from solution. For small molecules the entire molecule can be considered as either attached to the surface or free to migrate in the solution. The surface area occupied per molecule and the thickness of the adsorbed layer, as for example in the case of fatty acids, is between the limits for the molecule lying flat on the surface and the molecule with one end attached and at right angles to the surface. If the molecule is small, this difference may not be very large. Such molecules may be readily desorbed, and the breaking of a single bond releases the molecule into the solution. The amount of material adsorbed is directly related to the surface coverage and the kinetics of the adsorption frequently can be readily obtained using well known and verified kinetic treatments.

A far more complicated situation exists for polymer adsorption. The polymer molecule is probably initially adsorbed at one location, much in the same manner as would a small molecule. If it is not immediately desorbed, it is possible that the molecule may then uncoil with a high fraction of its segments adsorbing on the surface directly resulting in a relatively flat adsorbed film with each polymer molecule attached at many locations. Such a conformation has been proposed theoretically for certain polymer-surface-solvent interactions [5, 6]. Some experimental evidence has been interpreted to indicate such an arrangement [7, 8].

The adsorbed polymer molecule may also remain in a conformation closer to that of the molecule in solution. This would result in a relatively thick, highly swollen film with each molecule attached at a number of locations. The thickness of this layer would depend on the number of attachments per molecule and the distribution of these attachments. This type of final conformation has also been proposed theoretically [9].

It appears reasonable to expect that interactions between adsorbing polymer molecules would restrict the surface area available to each molecule and therefore the number of attachments per molecule. The number of such attachments will depend on the rate at which possible additional attachments are made by the adsorbed molecule compared to the rate at which available surface area is occupied by new molecules or neighboring adsorbed molecules. The rate of attachment of new molecules is, of course, proportional to the solution concentration, and the number of attachments per molecule would be dependent, in part, on the concentration of polymer in the solution. Therefore, for all the solution concentrations used in our study, the adsorbent surface is considered to be completely covered when there is no further adsorbance with time for a given concentration. The differences in the adsorbance with solution concentration result from differences in the conformation of the adsorbed molecule. The higher the solution concentration, the fewer the number of attachments per molecule, until a limiting adsorbance is attained.

This mechanism of polymer adsorption is consistent with the results reported here. The time required to reach plateaus in the adsorption curves given in figures 2 and 4 is not directly proportional to the solution concentrations. If only the rate of arrival of molecules at the surface were involved, the time required to attain equilibrium would be proportional to the solution concentration. Times to attain equilibrium from the more dilute solutions are less than would be expected from consideration of only the rate of arrival of polymer molecules. The initial rate of adsorption, as determined from the linear portions of the curves in figures 1 and 3 also is not directly proportional to the solution concentrations. The initial rate of adsorption is greater for the more concentrated solutions than would be expected from the same considerations.

The area available to a molecule approaching a surface from solution and therefore the opportunity for attachment of such a molecule is dependent on both the number of molecules on a surface and the area occupied by each molecule. As the solution concentration is decreased, the slower rate of arrival of the polymer molecules from solution may result in an increased number of attachments for each adsorbed molecule and consequently, more surface occupied per adsorbed molecule and less surface area available to approaching molecules from solution. This will result in fewer molecules being required for complete surface coverage as the solution concentration is decreased and therefore shorter times are required to reach maximum adsorption than what would be expected from solution concentration alone. The same reasoning can be applied to the initial rate of adsorption. As relatively more surface area is available per molecule in solution for the more concentrated solutions studied, the initial rate of adsorption is more rapid as the concentration is increased. Limiting values, not attained in this study, corresponding to the maximum adsorbance in an adsorption isotherm will, of course, occur.

Peterson and Kwei [8] applied the kinetic form of the Langmuir adsorption equation to their study of the rate of adsorption of poly (vinyl acetate) on chrome ferrotype surfaces. They treated surface coverage as proportional to the adsorbance, assumed no interaction between adsorbed polymer molecules, and obtained good agreement. Our data do not fit this equation. If our interpretation of the adsorption process is correct, we would not expect the Langmuir equation necessarily to fit all rate of adsorption data.

They also reported that at some concentrations the adsorbance reached a constant value after a few minutes, remained at this value for some time, and then ultimately reached a considerably higher adsorbance.

A somewhat similar initial plateau, termed a “resting period,” was observed by Jellinek and Northey [10] for the adsorption on charcoal of polystyrene from methyl ethyl ketone containing small quantities of water. Thorough drying of the solvent eliminated the resting period and decreased the rate of adsorption. The initial plateau was attributed to the more rapid adsorption of water compared to the larger polymer molecules, followed by eventual displacement of the adsorbed water by polymer. In our case, the adsorbance proceeded in a continuous manner, as observed in figures 2 and 4, until a constant adsorbance was attained, which did not change with additional time. No intermediate plateaus were observed.

An adsorbed molecule must be completely removed from a surface to detect desorption by the methods employed in this study. For this to occur all adsorbed portions of that molecule must be desorbed sufficiently long to permit the molecule to diffuse into the solution. Each adsorbed group can be considered to be repeatedly breaking and reestablishing its attachment. The opportunity for reattachment of the original group is dependent on the competition for the adsorption “site” by nonadsorbed groups of other adsorbed molecules or on “new” unadsorbed molecules from the solution.

On the basis of our point of view, the rate of desorption into solvent would be expected to depend on the adsorbance. As the adsorbance increases the number of attachments per molecule decreases, resulting in more easily removed molecules, Secondly, the competition for a freshly unoccupied site by neighboring absorbed molecules is higher as the adsorbance is increased, resulting in a larger probability that a detached group will stay detached, rather than readsorb at the same or another vacant site. Therefore, the rate of desorption would be larger as the adsorb ance increases. Polystyrene was found to desorb more rapidly as the adsorbance was increased, as shown in figures 6 and 7. As the adsorbance was decreased, the rate of desorption decreased until it was not detectable by our techniques. This is shown by curves F and G in figure 6 and, except for the first few seconds, by curve D in figure 7.

The higher molecular weight sample would have an opportunity for more attachments per molecule at the solution concentrations used than the lower molecular weight polymer. The rates of desorption for the lower molecular weight sample are larger than those for the higher molecular weight sample with similar adsorbance values. Hence the desorption behavior is qualitatively in accord with our views.

It might be expected that the average number of attachments per molecule, and the distribution of molecules with varying number of attachments would depend upon tne concentration of the solution from which the adsorption was carried out. This in turn should affect the rate of desorption. However, an inspection of curves C, D, and E in figure 6, which have approximately the same adsorbance although attained by exposure to different solution concentrations for different times, indicates that this is not the case, for the rates of desorption are very nearly the same. The rate of desorption into pure solvent appears to be dependent only on the adsorbance. This is also borne out by the behavior of curve A. To the extent that the rate of desorption is a measure of the conformation of the polymer on the surface and the number of segments attached, this indicates that in the desorption vessel, the conformation on the surface is the same for similar adsorbances.

This does not necessarily imply that at similar adsorbances on the adsorbance-time curves of figure 2 the conformation is also the same. Indeed, it is most likely not, for similar adsorbances occur at relatively different portions of the adsorbance-time curve at different solution concentration. But, when the slide with adsorbed polymer is placed in pure solvent, it appears that the polymer very rapidly adjusts its conformation so as to occupy essentially all the area available and hence the conformation becomes dependent only on the adsorbance. This readjustment of conformation, and consequent increase in the number of segments attached, apparently continues as the desorption proceeds. This is the most likely explanation of the behavior of curve A.

The thickness of adsorbed films of polystyrene, mol wt=76,000, was studied by means of ellipsometry [11, 12]. The solvent and adsorbent surface were the same as used in this study. The adsorbed layer was considered as an inhomogeneous film decreasing in polymer concentration, and hence refractive index, with distance from the surface. For such a distribution one may define a mean-square thickness as follows [13]:

$$<d^{2}>=\frac{\int_{0}^{\infty }x^{2}\left(n-n_{0}\right)dx}{\int_{0}^{\infty }\left(n-n_{0}\right)dx}$$

where *x* is the distance from the surface, *n* is the refractive index of the film and hence a function of *x*, and *n*_0_ is the refractive index of the solution. The root-mean-square thicknesses obtained in this manner were the same for a Gaussian, linear, or exponential variation of (*n–n*_0_) with *x*, and were approximately 50 Å for a solution concentration of 0.18 mg/ml and 115 Å for solution concentrations between 2 and 10 mg/ml. The average polymer concentration in the film was approximately 12 g/100 ml for most of the concentration range, with somewhat higher values for both the lower and higher solution concentrations.

The value of 115 Å is approximately the length of one component of the root-mean-square end to end distance at the theta temperature. Thus at high adsorbances the molecule has approximately the conformation of a random coil. At lower adsorbances, the conformation is more flattened, with presumably more attachments to the surface. The ellipsometry results are thus in qualitative agreement with the results reported here.

## Figures and Tables

The different symbols given for each concentration represent separate runs. Different slides used for each point.

The different symbols given for each concentration represent separate runs. Different slides used for each point.

The different symbols given for each concentration represent separate runs. Different slides used for each point.

The different symbols given for each concentration represent separate runs. Different slides used for each point.

—Solution concentration, 10^−3^ mg/ml. The same slides were used for each point.

Solid curves A, C, D, E, F, G obtained using different slides for each point.

Dashed curve B obtained using same slides for each point.

Solid curves A and D obtained using different slides for each point.

Dashed curves B and C obtained using same slide for each point.

## Footnotes

1 Figures in brackets indicate literature references at the end of this paper.

2 The ultracentrifugation of the samples was performed by Irwin Billick, National Bureau of Standards.
